# Effect of molecular flexibility on the rheological and filtration properties of synthetic polymers used as fluid loss additives in water-based drilling fluid

**DOI:** 10.1039/c9ra00038k

**Published:** 2019-03-14

**Authors:** Qi Chu, Ling Lin

**Affiliations:** State Key Laboratory of Shale Oil and Gas Enrichment Mechanisms and Effective Development Beijing 100101 China 190261829@qq.com; Sinopec Research Institute of Petroleum Engineering, Sinopec Beijing 100101 China; School of Chemistry and Chemical Engineering, Southwest Petroleum University Chengdu 610500 China; Oil & Gas Field Applied Chemistry Key Laboratory of Sichuan Province, Southwest Petroleum University Chengdu 610500 China

## Abstract

The effect of molecular flexibility on the rheological and filtration properties of synthetic polymers used as fluid loss additives in water-based drilling fluid was investigated. A new synthetic polymer (PAANS) comprising acrylamide (AM), 2-acrylamido-2-methyl-1-propane sulfonic acid (AMPS), *N*-vinyl-2-pyrrolidone (NVP) and potassium 2,5-dihydroxybenzenesulfonate (DHBS) was synthesized, in which phenyl groups were introduced in the backbone. Two other comparative polymers, PAAN and PAANS, were also prepared following the same synthesis procedure. PAAN comprises AM, AMPS and NVP, while PAANS consists of AM, AMPS, NVP and sodium 4-styrenesulfonate (SSS). PAAN, PAAND and PAANS were characterized by ^1^H NMR and elemental analysis, and the molecular weight was determined by static light scattering (SLS). The rheological properties, filtration properties and performance sustainability were investigated. Using a rheological properties measurement test, the apparent viscosity (AV), plastic viscosity (PV) and yield point (YP) of the Na-MMT/PAAND system at a concentration of 2.0% were 18.0 mP s, 12.0 mP s and 6.0 Pa, respectively, after a thermal aging test at 240 °C for 16 h. These values are much higher than those of the corresponding Na-MMT/PAAN and Na-MMT/PAANS systems. The API filtration loss volume (FL_API_) and high-temperature/high-pressure filtrate volume (FL_HTHP_) of the Na-MMT/PAAND system at a concentration of 2.0% were 12.0 mL and 30.0 mL, respectively, after a thermal aging test at 240 °C for 16 h. These values are much lower than those of the Na-MMT/PAAN and Na-MMT/PAANS systems. Compared with PAAN and PAANS, PAAND presents the best performance sustainability after multiple shearing and thermal aging tests. At the same temperature, the order of maintaining rheological performance and controlling the FL_API_ and FL_HTHP_ was PAAND > PAANS > PAAN in Na-MMT/PAANS-based drilling fluid at high temperature. Increasing the percentage of rigid monomers in the backbone was found to be conducive to maintaining the rheological stability and improving the filtration properties at high temperature. The control mechanism of fluid loss was investigated through adsorption tests using the method of thermal filtration, assessing particle size distribution on a laser diffraction particle size analyzer (LPSA) and examining filter cake morphologies using an environmental scanning electron microscope (ESEM). The results reveal that the introduction of rigid monomers into the synthetic polymer backbone can effectively improve the adsorption capacity of the polymer on the clay surface, obstruct the aggregation of clay particles, and improve the quality of filter cakes at high temperatures.

## Abstract 2

The effect of molecular flexibility on the rheological and filtration properties of synthetic polymers used as fluid loss additives in water-based drilling fluid was investigated.

## Graphical abstract

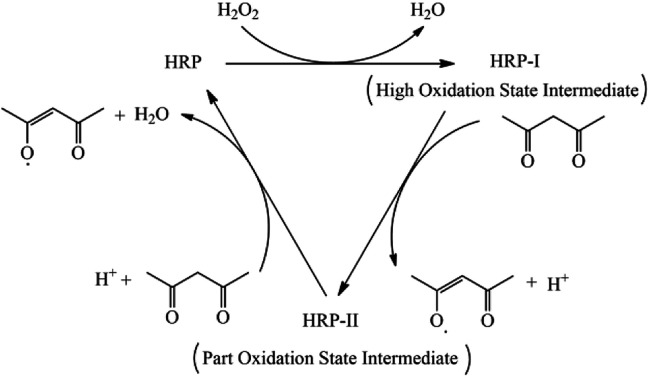

## Introduction

Drilling fluids, also referred to as “drilling muds”, play an important role in drilling operations. They perform essential tasks including lubricating the drilling tool and drill pipe that carries the tool, suspending and transporting formation cuttings to the surface for screening and disposal, holding the cuttings in suspension in the event of a shutdown in the drilling and pumping of the drilling fluid, and counterbalancing formation pressures to prevent the inflow of gas, oil or water from permeable rocks, which may be encountered at various levels in the drilling process.^1–4^ One of the most important functions of drilling fluid, the filtration performance, is to form an impervious filter cake on the wall of the wellbore, prevent water leakage, block pressure transport from the wellbore to the formation, and maintain the stability of the well wall. Adding a fluid loss additive is the best way to improve the filtration performance of drilling fluid.^5–7^ Currently, there are two kinds of fluid loss additives: natural polymer compounds (*e.g.*, starch, xanthan gum, carboxymethyl cellulose and polyanionic cellulose lignite) and synthetic polymers.^8–13^ However, as oil and gas exploration is increasingly focused on deep formations, synthetic polymers, such as 2-acrylamido-2-methylpropane sulfonic acid (AMPS) multi-copolymers, are most widely used as fluid loss additives in drilling fluid because of their high temperature resistance. At present, many modifications of high temperature fluid loss additives are based on AMPS multi-copolymers.^14–16^

In recent decades, research has considerably enriched our knowledge about the mechanisms behind how these synthetic polymers prevent fluid loss. It has shown that synthetic polymers as fluid loss additives contain at least two groups: an adsorption group and a hydration group. Adsorption groups, such as –OH, –SiOH, –CONH_2_ and cationic groups, are used to enhance the interaction between the polymer and bentonite. The polymeric fluid loss additive can therefore better adsorb on the bentonite surface *via* hydrogen bonding, coordination bonding or ionic bonding to raise the ζ potential and hydrated shell thickness of the bentonite particles. Hydration groups, such as –COO^−^ and –SO_3_^−^, are used to improve the dispersion properties of bentonite. They reduce the attractions between bentonite particles by electrostatic stabilization, strengthen the bentonite structure, and help to plug the filter cake holes.^17,18^ Therefore, recent efforts to modify polymeric fluid loss additives have mainly been focused on adsorption and hydration groups. However, it is worth noting that the temperature resistance of a polymeric fluid loss additive obtained by changing the types and proportions of adsorbed and hydrated groups in the molecule rarely exceeds 230 °C. In order to further improve the temperature resistance of polymeric fluid loss additives, other molecular modification methods must be considered.

In other polymer fields, since Hill and Walker first pointed out that the incorporation of aromatic segments into a polymer generally results in a noticeable increase in its thermal stability, considerable research has been conducted to improve the rigidity of molecules by incorporating or grafting aromatic units.^19–21^ Many of these aromatic polymers have been commercialized, such as aromatic polyamides, polyimides, polyesters, polysulfones, and heterocyclic polymers.^22–27^ This method can also be applied to the modification of polymeric fluid loss additives. Polymeric fluid loss additives with high temperature resistance usually tend to contain more aromatic units in their structure. Wan *et al.* used the inverse micro-emulsion polymerization method to synthesize copolymers of different hydrophilic monomers, such as acrylamide (AM), acrylic acid (AA) and sodium 4-styrenesulfonate (SSS). The maximum thermal stability of the AM/AA/SSS copolymer was found to be higher than 230 °C.^28^ Ma *et al.* used the free radical polymerization method to synthesize copolymers of different hydrophilic monomers, such as AM, AMPS, diallyl dimethyl ammonium chloride (DMDAAC) and vinylphenyl sulfonate (VPS). In their study, the API filtration loss volume (FL_API_) of freshwater drilling fluid containing 1.25% polymeric fluid loss additive was only 4.8 mL, and it was 19.2 mL after aging at 200 °C for 16 h.^29^

This paper concerns the investigation of the relationship between the flexibility and thermal stability of polymeric fluid loss additives, in order to enhance the temperature resistance of polymeric fluid loss additives by introducing rigid monomers into their molecular structures. In this study, SSS and potassium 2,5-dihydroxybenzenesulfonate (DHBS) were chosen as the rigid monomers, which were introduced into an AM/AMPS/NVP copolymer. SSS was used as a rigid monomer for the construction of part of the side chain of AM/AMPS/NVP/SSS. DHBS was used as a rigid monomer for the construction of part of the main chain of AM/AMPS/NVP/DHBS. Three different kinds of copolymers were synthesized to compare the effect of the structure on the thermal stability of rheological and filtration properties.

## Experimental

### Materials

Pristine sodium montmorillonite (Na-MMT) was obtained as a commercial product from Xinjiang Xiazijie bentonite Co., Ltd., and it had a cation exchange capacity (CEC) of 118.4 mequiv./100 g.

Acrylamide (AM), 2-acrylamido-2-methyl-1-propane sulfonic acid (AMPS), *N*-vinyl-2-pyrrolidone (NVP), sodium 4-styrenesulfonate (SSS), and potassium 2,5-dihydroxybenzenesulfonate (DHBS) were purchased from Shanghai Aladdin Bio-chem Technology Co., Ltd., and were used without further purification. The initiator was horseradish peroxidase (HRP, enzyme activity ≥ 200 units per mg solid), which was purchased from Shanghai Aladdin Reagent Co. Hydrogen peroxide (H_2_O_2_, 10.0%, w/v) from Chengdu Huabo Chemical Reagent Co. and acetylacetone (ACAC), dipotassium hydrogen phosphate, sodium hydroxide, 1,4-dioxane, acetone and ethanol from Chengdu Kelong Chemical Reagent Co. of analytical grade were used as catalyzer, neutralizer and solvent respectively.

### Synthesis

The polymerization was carried out in a four-necked round bottom flask equipped with a stirrer, thermometer, nitrogen gas inlet and condenser. Firstly, a buffer solution of pH = 6.5 was obtained by diluting 1.36 g dipotassium hydrogen phosphate with 0.1 mol L^−1^ sodium hydroxide solution of 30.4 mL with water to 200 mL. Then, 100 mg HRP was dissolved in 50 mL water and stored at 4 °C. Secondly, AM, AMPS, NVP and DHBS were added to 1,4-dioxane, and the mixture was then transferred into the above flask. Thirdly, 200 mL buffer solution was added to the reaction flask after 30 min of deoxygenation with N_2_. After mixing, the HRP solution and ACAC were added and then the mixture was heated to 50 °C. The reaction was stopped after 12 h. During the whole polymerization process, H_2_O_2_ solution was injected gradually into the flask through a plastic septum by using a syringe. Finally, the product was cut into small pieces by hand and vacuum-dried at 75 °C for 24 h and stored in desiccators.

**Fig. 1:**
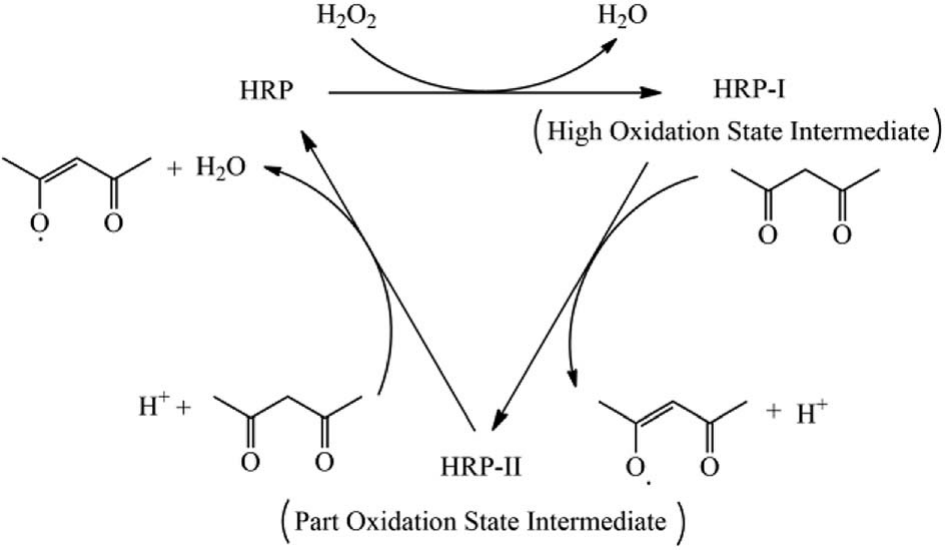
The catalytic mechanism of HRP.

**Fig. 2:**
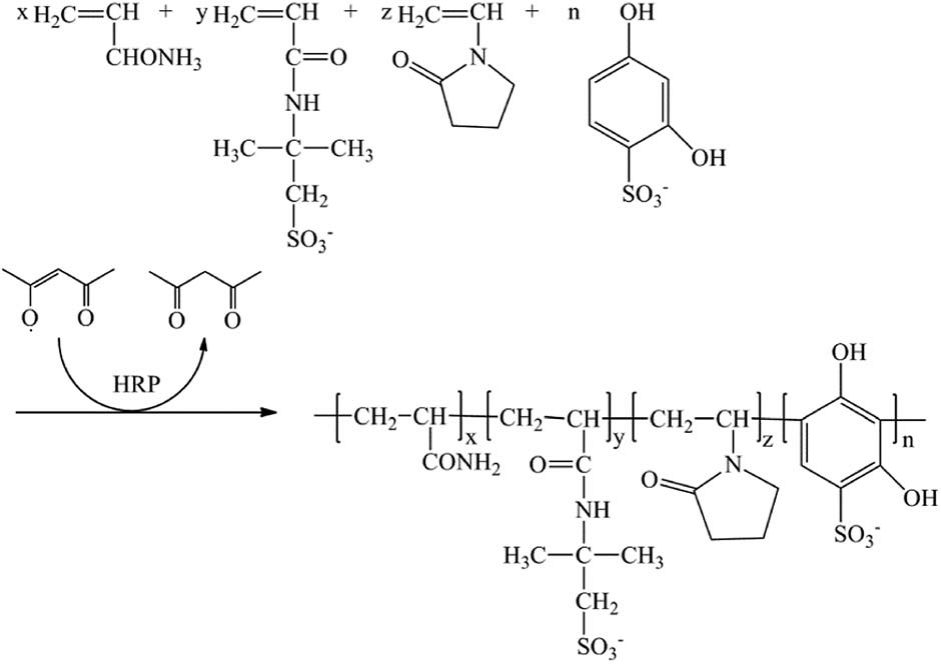
The copolymerization schematic diagram of PAAND.

The molar ratio of AM, AMPS, NVP and DHBS was 60 : 20 : 12 : 8. The total weight percent of the four monomers in 1,4-dioxane was 10.0%. The weight of the HRP solution compared with the monomers was 0.02%. The weight of ACAC and H_2_O_2_ solution compared with the monomers was 0.05% and 0.08%, respectively. Poly(AM/AMPS/NVP/DHBS) was named PAAND. Poly(AM/AMPS/NVP) and poly(AM/AMPS/NVP/SSS) were named PAAN and PAANS, respectively, which were synthesized and dried under the same polymerization conditions, but the DHBS monomer was not added in PAAN and the DHBS monomer was replaced with SSS in PAANS. The catalytic mechanism of HRP and copolymerization schematic diagram of PAAND are shown in Fig. 1 and 2.

### Characterization

PAAN, PAAND and PAANS were precipitated with ethanol for 12 h and washed three times with acetone, thus removing the unreacted monomer. The products were dried at 40 °C. Then, PAAN, PAAND and PAANS were removed from the precipitated sample by Soxhlet extraction with a 60 : 40(v/v) mixture of ethylene glycol and acetic acid. Finally, PAAN, PAAND and PAANS were washed with ethanol, and were dried in a vacuum oven at 40 °C until the samples reached a constant weight.

^1^H nuclear magnetic resonance (^1^H NMR) analysis was used to determine the molecular structures and functional groups. ^1^H NMR analysis was conducted on an Avance II 400 MHz NMR spectrometer (Bruker Instrument Co. Ltd., Switzerland) to obtain proton nuclear magnetic resonance spectra. D_2_O was used for the field-frequency lock, and the observed ^1^H chemical-shifts were reported in parts per million (ppm). The elemental analysis of PAAN, PAAND and PAANS was conducted on an EA 2400II elemental analyzer (PerkinElmer Instrument Co. Ltd., USA) to determine the carbon, nitrogen, oxygen and sulfur content. Determination of the molecular weight distributions of PAAN, PAAND and PAANS was conducted through static light scattering (SLS) measurements (Wyatt Technology Inc., Canada) using a Gs-As laser (658 nm and 40 mW).

### Performance evaluation

Drilling fluid was prepared according to American Petroleum Institute (API) specifications. The water-based drilling fluid (4.0% prehydrated Na-MMT) was made up by maintaining the Na-MMT to H_2_O weight ratio at 4 : 100. Prior to use, the water-based drilling fluids were aged for 24 h at room temperature to hydrate.

A certain amount (w/v) of PAAN, PAAND and PAANS was dissolved in the water-based drilling fluid with stirring at 10 000 rpm for 20 min. Thermal aging tests of Na-MMT/PAAN, Na-MMT/PAAND and Na-MMT/PAANS were carried out in a GH-3-type rolling oven through hot rolling at an appointed temperature (160, 170, 180, 190, 200, 210, 220, 230, 240, 250 and 260 °C) for 16 h. Fluid property tests were performed before and after the thermal aging tests.

The rheological properties of the treated water-based drilling fluids were measured using a DNN-Z6 type six-speed rotating viscometer (Qingdao Haitongda Special Instrument Co., Ltd., China). Rheological parameters such as apparent viscosity (AV), plastic viscosity (PV) and yield point (YP) can be calculated from 300 and 600 rpm readings (*Φ*_300_ and *Φ*_600_) as per eqn (1)–(3).

1 AV = 0.5*Φ*_600_ (mPa s)

2 PV = *Φ*_600_ − *Φ*_300_ (mPa s)

3 YP = 0.5(2*Φ*_300_ − *Φ*_600_) (Pa)

The ratio of YP and PV (RYP) is an important rheological parameter. It is a measure of the shear thinning behavior of drilling fluids. A high ratio indicates outstanding carrying efficiency and lower settling velocity of cuttings. If the gauge hole is not maintained and the diameter of the borehole increases, a fluid with a high RYP is desirable.^30,31^

The FL_API_ of water-based drilling fluid was measured using a ZNZ-D3 type medium-pressure filtration apparatus (Qingdao Haitongda Special Instrument Co., Ltd., China). The high-temperature/high-pressure filtrate volume (FL_HTHP_) was determined with a GGS42 type high-temperature/high-pressure filtration apparatus (Qingdao Jiaonan Tongchun Machinery Petroleum instrument Ltd., China). FL_API_ and FL_HTHP_ were loaded on the filter press equipped with a filter paper under a fixed pressure of 0.7 MPa and 3.5 MPa, respectively. The results were recorded after 30 min, as recommended by American Petroleum Institute (API) specifications.

In a drilling operation, during the process of drilling fluid being mechanically sheared by the mud pump, impacting the bottom hole and scouring the well wall, some chemical bonds of the polymer in the fluid become broken, negatively impacting the performance of the drilling fluid. Agents with good performance sustainability can not only reduce the material consumption, but also help to reduce the maintenance frequency of the drilling fluid. Therefore, it is necessary to evaluate the performance sustainability of agents.

First, the Na-MMT/PAAN, Na-MMT/PAAND and Na-MMT/PAANS systems were stirred at a speed of 10 000 rpm for 20 min using a TD250-2-type high-speed mechanical blender (Beijing Kelishi Petroleum instrument, China) to simulate the process of high-speed shear damage to the internal structure of the drilling fluid during injection of the mud pump, and then to determine its FL_API_ and FL_HTHP_. Second, the Na-MMT/PAAN, Na-MMT/PAAND and Na-MMT/PAANS systems were placed in a GH-3-type rolling oven where they underwent hot rolling at appointed temperatures for 4 h to simulate the effect of the high temperature at the bottom of the well on the composition of the drilling fluid. The Na-MMT/PAAN, Na-MMT/PAAND and Na-MMT/PAANS systems were stirred at a speed of 10 000 rpm for 20 min again, and then FL_API_ and FL_HTHP_ were determined. The performance sustainability of the drilling fluid during the whole circulation process in drilling operation was evaluated by repeating step 2.

### Mechanism analysis

The amount of PAAN, PAAND and PAANS adsorbed onto Na-MMT in water-based drilling fluid was measured using Vario TOC total organic carbon analysis (Elementar Analysensysteme GmbH, Germany) using thermal filtration. This was done following the procedure previously described by Chu *et al.*^32^ The method of thermal filtration can accurately reflect the adsorption amount of the polymer molecules under high temperature conditions.

The particle size distribution was determined using a Mastersizer 3000 ultra-high speed intelligent laser diffraction particle size analyzer (LPSA, Malvern Instruments Ltd., Britain).

The fresh filter cake from the fluid loss test was cut horizontally into two equal halves. The surface of a fragment that was inside the filter cake was investigated using a Quanta 450 environmental scanning electron microscope (ESEM, FEI Co., USA) with an Everhart–Thornley secondary electron detector (accelerating potential 25.0 kV at 3.0 °C and 6.8 mbar). The ESEM allows for the viewing of the wet filter cakes in their original and unaltered state.

## Results and discussion

### Characterization

**Fig. 3:**
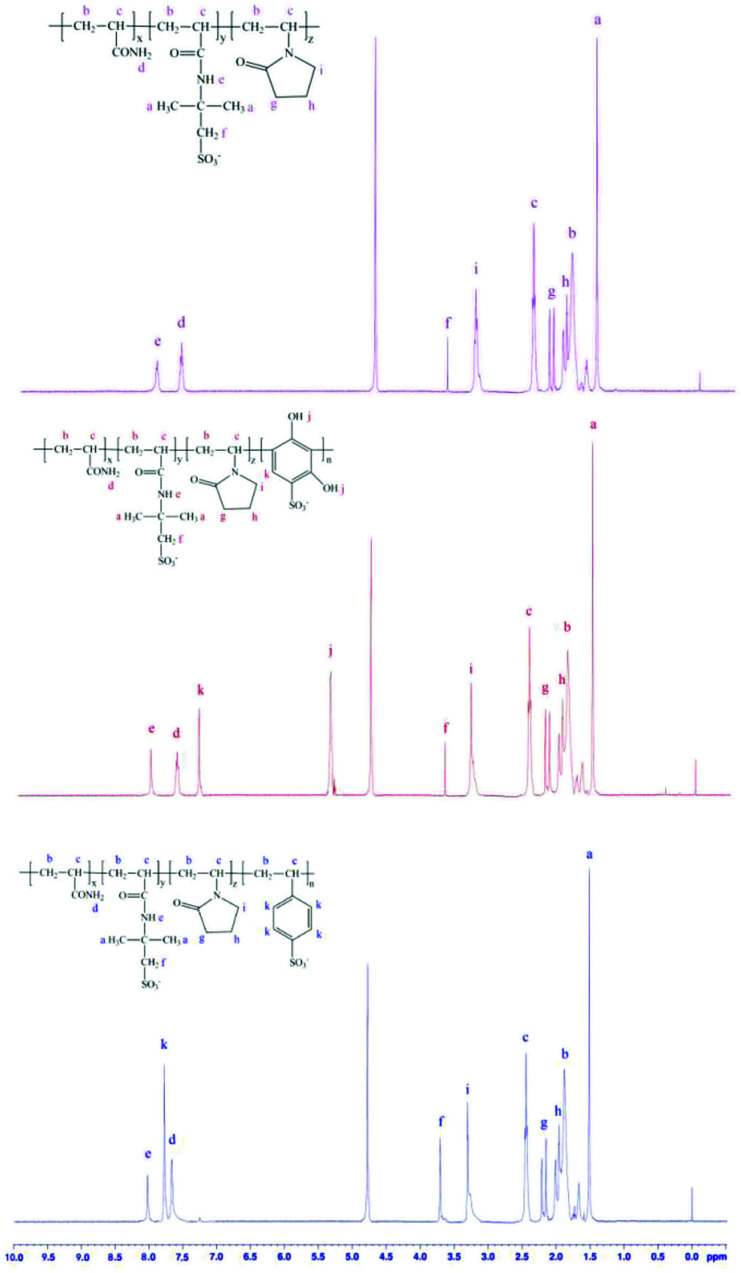
^1^H NMR spectra of PAAN, PAAND and PAANS.

The ^1^H NMR spectra of PAAN, PAAND and PAANS are shown in Fig. 3. In Fig. 3, several characteristic peaks of different monomers can be seen in the spectra of PAAN, PAAND and PAANS. For PAAN, a peak for –CH_3_ in AMPS can be seen at 1.52 ppm. Peaks for –CH_2_–CH–CO– and –CH–CO– in the backbone of the polymer are present in the regions of 1.68–2.08 ppm and 2.43–3.23 ppm, respectively. Peaks for –CO–NH_2_ in AM and –CO–NH– in AMPS can be observed at 7.66 and 8.03 ppm, respectively. The peak at 3.71 ppm is for –CH_2_–SO_3_^−^ in AMPS. Peaks for the ring structure protons of NVP are present at 1.91, 2.18 and 3.29 ppm. Compared with PAAN, typical chemical-shift values of protons in PAAND for –OH and phenyl of DHBS were observed at 5.35 ppm and 7.30 ppm, respectively, and a typical chemical-shift value of protons in PAANS for phenyl of SSS was observed at 7.78 ppm. The ^1^H NMR spectrum affirmed the successful copolymerization of the monomers.

The weight-average molecular weight (*M*_w_) and number-average molecular weight (*M*_n_) were obtained from SLS analysis in 0.1 mol L^−1^ NaCl solution. The composition, *M*_w_ and *M*_n_ of PAAN, PAAND and PAANS are shown in Table 1. Table 1 illustrates that the polymer molar composition of DHBS in PAAND was lower than the corresponding monomer feed composition, but that of SSS in PAANS was higher than the corresponding monomer feed composition, which was probably due to the reactivity ratio difference of DHBS compared to other monomers. PAAN, PAAND and PAANS have smaller *M*_w_ and *M*_n_ values, indicating that DHBS and SSS have an insignificant effect on molecular weight.

**Table 1:** Composition and molecular weight of PAAN, PAAND and PAANS

Sample	Feed molar composition (AM/AMPS/NVP/DHBS) or (AM/AMPS/NVP/SSS)	Elemental composition (%)				Polymer molar composition (AM/AMPS/NVP/DHBS) or (AM/AMPS/NVP/SSS)	*M*_w_ (×10^4^, g mol^−1^)	*M*_n_ (×10^4^, g mol^−1^)
		C	N	O	S			
PAAN	60 : 20 : 12 : 0	46.2925	12.4509	24.0542	6.5682	58.50 : 21.20 : 12.30 : 0	7.9931	8.1321
PAAND	60 : 20 : 12 : 8	44.4721	10.6686	25.3449	7.3727	55.72 : 23.63 : 14.55 : 6.10	7.7600	7.8540
PAANS	60 : 20 : 12 : 8	46.3432	10.5410	23.9306	7.9465	57.39 : 21.02 : 12.63 : 8.96	8.0564	8.1719

In order to investigate the influence of the percentage of DHBS in PAAND and that of SSS in PAANS on the rheological and filtration properties in water-based drilling fluid, a series of polymers (PAAND and PAANS) containing rigid monomers (DHBS and SSS) with different ratios were synthesized using a similar procedure. The products are abbreviated as PAAND-1 to PAAND-6 and PAANS-1 to PAANS-6, respectively. The main compositions are listed in Table 2.

**Table 2:** Composition of PAAND-1 to PAAND-6 and PAANS-1 to PAANS-6

Sample	Feed molar composition (AM/AMPS/NVP/DHBS) or (AM/AMPS/NVP/SSS)	Polymer molar composition (AM/AMPS/NVP/DHBS) or (AM/AMPS/NVP/SSS)
PAAND-1	60 : 20 : 12 : 4	59.28 : 24.02 : 13.74 : 2.96
PAAND-2	60 : 20 : 12 : 6	56.96 : 23.83 : 14.17 : 5.04
PAAND-3	60 : 20 : 12 : 10	58.74 : 21.11 : 11.97 : 8.18
PAAND-4	60 : 20 : 12 : 12	57.91 : 20.52 : 11.44 : 10.13
PAAND-5	60 : 20 : 12 : 14	56.32 : 20.20 : 11.36 : 12.12
PAAND-6	60 : 20 : 12 : 16	55.59 : 20.01 : 11.02 : 13.38
PAANS-1	60 : 20 : 12 : 4	63.21 : 20.51 : 12.11 : 4.17
PAANS-2	60 : 20 : 12 : 6	60.63 : 21.05 : 12.14 : 6.18
PAANS-3	60 : 20 : 12 : 10	57.69 : 20.75 : 12.00 : 9.56
PAANS-4	60 : 20 : 12 : 12	56.21 : 20.36 : 11.85 : 11.57
PAANS-5	60 : 20 : 12 : 14	54.75 : 20.14 : 11.61 : 13.50
PAANS-6	60 : 20 : 12 : 16	53.07 : 20.00 : 11.54 : 15.39

### Rheological properties

Table 2 shows the effect of PAAN, PAAND and PAANS concentration on the rheological characteristics of the water-based drilling fluid after thermal aging tests at 200 °C and 240 °C. After adding PAAN, AV, PV and YP of the Na-MMT-based drilling fluid increase. Compared with PAAN, the rheological parameter values of the Na-MMT-based drilling fluids containing PAAND or PAANS were much higher after treatment at the same thermal aging temperature, although *M*_w_ and *M*_n_ of PAAND and PAANS were similar to that of PAAN. The rheological parameter values of PAAND and PAANS were similar after thermal aging test at 200 °C, but differentiated when the thermal aging temperature reached 240 °C. When the concentration was 2.0%, AV, PV and YP of the Na-MMT/PAAND system were 18.0 mP s, 12.0 mP s and 6.0 Pa, respectively, after thermal aging tests at 240 °C. Under the same conditions, AV, PV and YP of the Na-MMT/PAAN system were 7.0 mP s, 5.0 mP s and 2.0 Pa, respectively, and AV, PV and YP of the Na-MMT/PAANS system were 8.5 mP s, 6.0 mP s and 2.5 Pa, respectively. The results indicate the Na-MMT-based drilling fluid treated with PAAND has excellent rheological properties. Hence, it is reasonable to believe that synthetic polymers with rigid monomers in their backbone have higher temperature resistance than those with rigid monomers in their side chains.

When the concentration was 2.0%, the Na-MMT-based drilling fluids treated with PAAN, PAAND and PAANS were hot rolled at different temperatures for 16 h, respectively. Rheological parameters were measured after the thermal aging tests (Table 3). The results are shown in Table 4.

**Table 3:** Effect of concentration on the rheological properties of PAAN, PAAND and PAANS in Na-MMT-based drilling fluid after thermal aging tests

Fluid formulation	After thermal aging at 200 °C			After thermal aging at 240 °C		
	AV (mPa s)	PV (mPa s)	YP (Pa)	AV (mPa s)	PV (mPa s)	YP (Pa)
Na-MMT-based drilling fluid	4.0	3.5	0.5	3.5	3.5	0
+0.5% PAAN	9.0	6.0	3.0	5.0	4.5	0.5
+0.5% PAAND	12.0	7.0	5.0	10.0	6.0	4.0
+0.5% PAANS	12.0	7.0	5.0	6.0	5.0	1.0
+1.0% PAAN	12.5	8.0	4.5	5.0	4.5	0.5
+1.0% PAAND	15.0	9.0	6.0	12.0	7.5	4.5
+1.0% PAANS	15.0	9.0	6.0	7.0	5.5	1.5
+1.5% PAAN	16.5	10.0	6.5	5.5	4.5	1.0
+1.5% PAAND	19.5	11.5	8.0	14.5	9.0	5.5
+1.5% PAANS	20.0	12.0	8.0	7.5	6.0	1.5
+2.0% PAAN	21.0	14.0	7.0	7.0	5.0	2.0
+2.0% PAAND	27.5	16.0	11.5	18.0	12.0	6.0
+2.0% PAANS	27.0	16.0	11.0	8.5	6.0	2.5

**Table 4:** Effect of thermal aging temperature on the rheological properties of PAAN, PAAND and PAANS in Na-MMT-based drilling fluid

Fluid formulation	Thermal aging temperature (°C)	AV (mPa s)	PV (mPa s)	YP (Pa)
Na-MMT-based drilling fluid + 2.0% PAAN	160	30.0	17.0	13.0
	180	27.0	16.0	11.0
	200	21.0	14.0	7.0
	220	12.0	8.0	4.0
	240	7.0	5.0	2.0
	260	2.0	2.0	0
Na-MMT-based drilling fluid + 2.0% PAAND	160	32.5	18.5	14.0
	180	29.5	17.0	12.5
	200	27.5	16.0	11.5
	220	22.0	12.0	10.0
	240	18.0	12.0	6.0
	260	6.5	5.0	1.5
Na-MMT-based drilling fluid + 2.0% PAANS	160	33.0	18.0	15.0
	180	29.0	17.0	12.0
	200	27.0	16.0	11.0
	220	17.0	11.0	6.0
	240	8.5	6.0	2.5
	260	3.0	2.0	1.0

The information in Table 4 can be summarized as follows. First, the rheological parameters of the Na-MMT/PAAN, Na-MMT/PAAND and Na-MMT/PAANS systems gradually deceased with increasing temperature from 160 °C to 260 °C. Second, compared with PAAN, the rheological parameters of the Na-MMT-based drilling fluids treated with PAAND or PAANS were much higher at the same temperature, meaning that PAAND and PAANS have excellent thermal stability. Third, the rheological parameters of PAAND and PAANS were similar when the thermal aging temperature was less than 200 °C, but when the thermal aging temperature exceeded 200 °C, the rheological parameters of PAAND were significantly higher than those of PAANS. Additionally, when the thermal aging temperature was raised to 260 °C, AV, PV and YP of the Na-MMT/PAAND system were 6.5 mP s, 5.0 mP s and 1.5 Pa, and those of the Na-MMT/PAANS system were 3.0 mP s, 2.0 mP s and 1.0 Pa, respectively, indicating that the synthetic polymer modified with rigid monomers as a fluid loss additive may have failed due to thermal degradation. This phenomenon can be explained as follows. The rigid monomer distributed in the backbone or side chain of the molecule can effectively reduce the vibration degree of freedom of the molecular chain; that is, the rigid monomer reduces the movement velocity of the molecule at high temperature and reduces the incidence of the unwinding of the polymer. The interaction force between polymer molecules and between the polymer and Na-MMT is improved, and the stability of the 3D network structure of the drilling fluid is improved, which makes the polymer show strong thermal stability in water-based drilling fluid. Compared with the modification of the molecular side chain by rigid monomers, adding rigid monomers to the backbone can reduce the thermal movement of the molecule at high temperature to a certain extent, thus leading to better high temperature resistance in water-based drilling fluid.

The influence of the percentage of rigid monomers in the polymer molecule on the rheological properties of the series of polymers in Na-MMT-based drilling fluid (at a concentration of 2.0%) after thermal aging tests at 200 °C and 240 °C was investigated, and the results are shown in Table 5. It is apparent that the rheological parameters gradually increase with increasing rigid monomer percentage in the polymer after thermal aging tests at 200 °C. By comparing the rheological parameters of the series of polymers in Na-MMT-based drilling fluid, it was found that there are no significant differences in introducing rigid monomers into the backbone compared to the side chains in improving the rheological stability of water-based drilling fluid. When the thermal aging temperature reached 240 °C, for the PAAND-type series of polymers, the rheological parameters slightly increased with the increase in the percentage of rigid monomer, and were significantly higher than that of PAAN, showing good rheological stability. However, for the PAANS-type series of polymers, the rheological parameters were similar to those of PAAN, indicating that if rigid monomers are introduced into the side chain, the stability of improving the rheological properties of water-based drilling fluid is not significant. This is mainly due to the introduction of rigid monomers, especially their introduction into the backbone, which is more beneficial to improving the thermal stability of a polymeric fluid loss additive.

**Table 5:** Effect of rigid monomer percentage on rheological properties in Na-MMT drilling fluid after thermal aging tests

Sample	After thermal aging at 200 °C			After thermal aging at 240 °C		
	AV (mPa s)	PV (mPa s)	YP (Pa)	AV (mPa s)	PV (mPa s)	YP (Pa)
PAAN	21.0	14.0	7.0	7.0	5.0	2.0
PAAND-1	21.5	14.5	7.0	7.5	5.0	2.5
PAAND-2	23.5	15.0	8.5	11.0	7.0	4.0
PAAND	27.5	16.0	11.5	18.0	12.0	6.0
PAAND-3	29.5	17.5	12.0	19.0	12.5	6.5
PAAND-4	30.0	18.0	12.0	19.5	12.5	7.0
PAAND-5	31.0	18.5	12.5	19.5	12.5	7.0
PAAND-6	32.0	19.0	13.0	20.5	13.0	7.5
PAANS-1	21.5	14.5	7.0	7.0	5.0	2.0
PAANS-2	23.0	15.0	8.0	8.0	5.5	2.5
PAANS	27.0	16.0	11.0	8.5	6.0	2.5
PAANS-3	29.0	17.0	12.0	10.0	7.0	3.0
PAANS-4	29.5	17.5	12.0	10.0	7.0	3.0
PAANS-5	29.5	17.5	12.0	10.0	7.0	3.0
PAANS-6	30.5	18.0	12.5	10.5	7.5	3.0

### Filtration properties

**Fig. 4:**
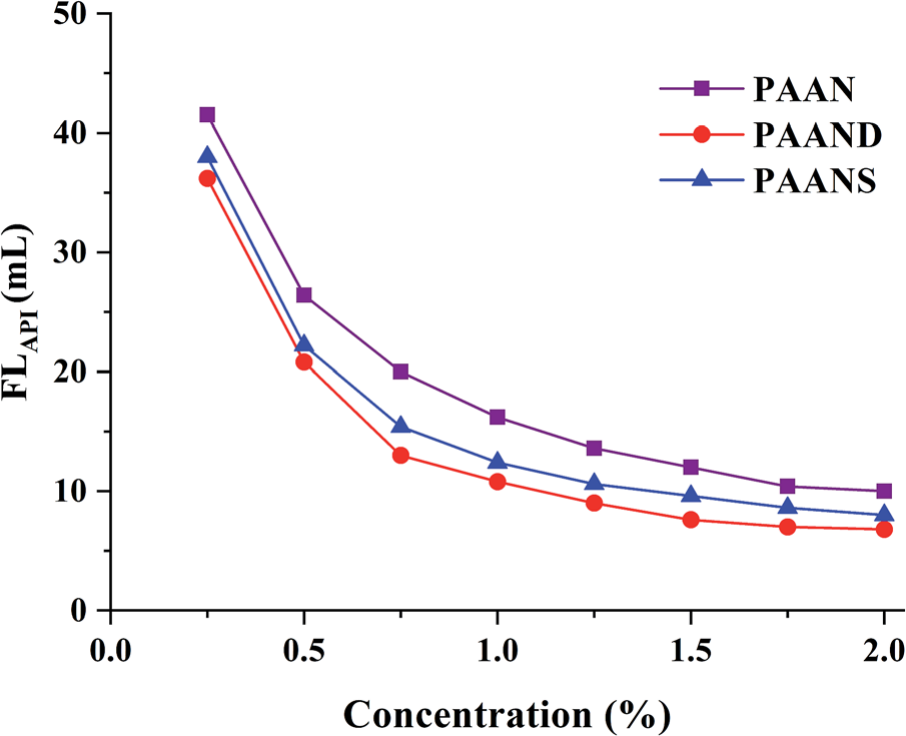
Effect of concentration on FL_API_ of PAAN, PAAND and PAANS in Na-MMT-based drilling fluid after thermal aging tests at 200 °C.

**Fig. 5:**
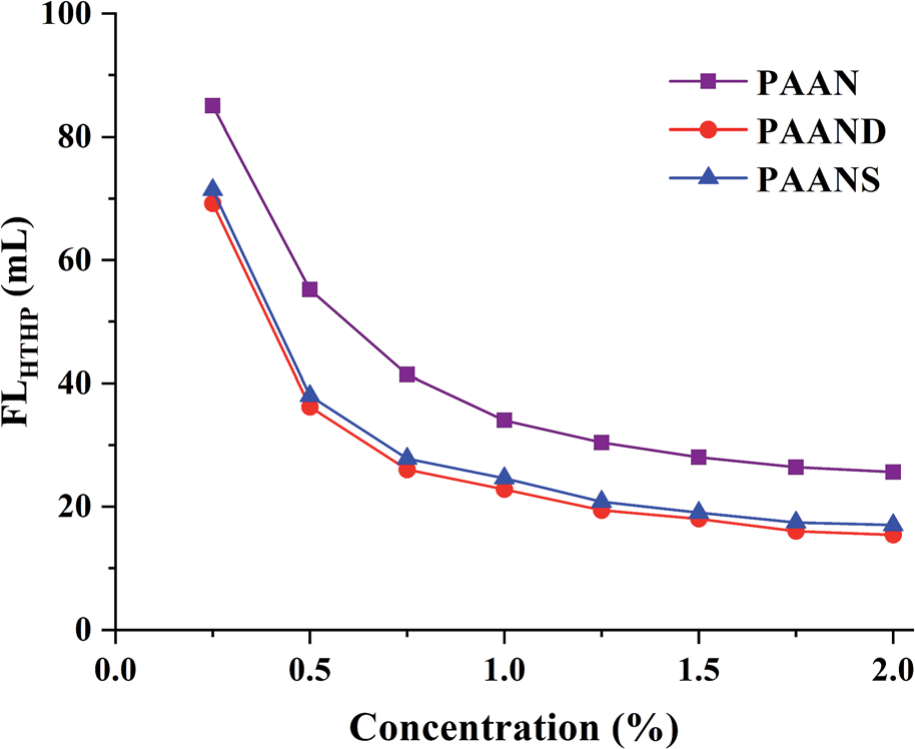
Effect of concentration on FL_HTHP_ of PAAN, PAAND and PAANS in Na-MMT-based drilling fluid after thermal aging tests at 200 °C.

A series of filtration tests for PAAN, PAAND and PAANS were conducted in Na-MMT-based drilling fluid after thermal aging at 200 °C for 16 h. In Fig. 4 and 5, we can see that with the increase of the concentration, the FL_API_ and FL_HTHP_ of the Na-MMT/PAAN, Na-MMT/PAAND and Na-MMT/PAANS systems decreased gradually, and when the concentration was higher than 1.5%, the decreasing trend of drilling fluid filtration slowed down. PAAND showed the best performance in controlling the FL_API_ and FL_HTHP_. When the concentration ranged from 0.25% to 2.0%, FL_API_ of the Na-MMT/PAAND system decreased from 36.2 mL to 6.8 mL, and FL_HTHP_ of the Na-MMT/PAAND system decreased from 69.2 mL to 15.4 mL.

At the same concentration, the FL_API_ and FL_HTHP_ of the Na-MMT/PAAND system were slightly lower than those of the Na-MMT/PAANS system, indicating that it is more advantageous to improve the filtration performance by introducing rigid monomers in the backbone of the polymer molecules than in the side chains. Compared with PAAN and PAANS, the combination formed between PAAND and clay has stronger stability, which thickens the hydration film on the surface of clay particles and prevents the aggregation of clay particles into large particles due to collision, thus helping to maintain the content of fine particles in the drilling fluid, forming a mud cake with low permeability and reducing filtration loss.

**Fig. 6:**
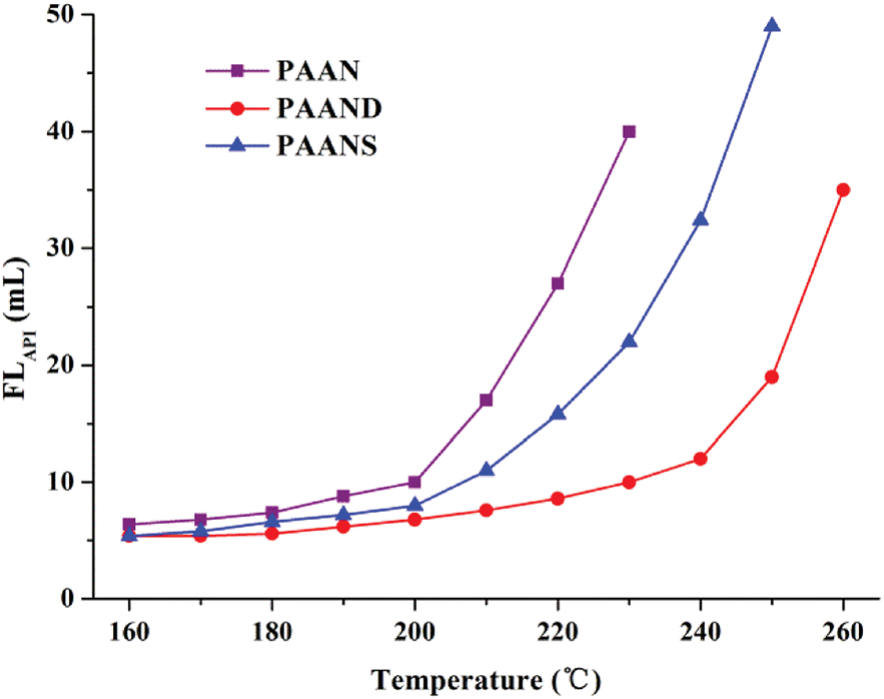
Effect of temperature on FL_API_ of PAAN, PAAND and PAANS in Na-MMT-based drilling fluid after thermal aging tests.

**Fig. 7:**
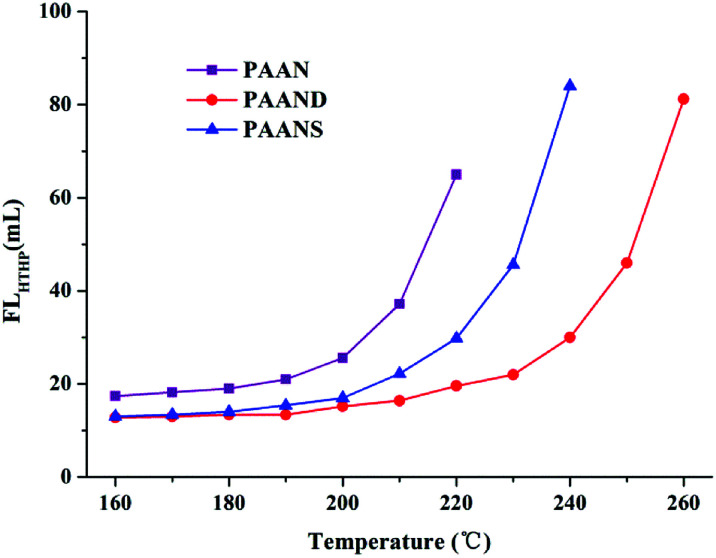
Effect of temperature on FL_HTHP_ of PAAN, PAAND and PAANS in Na-MMT-based drilling fluid after thermal aging tests.

The effect of temperature on FL_API_ and FL_HTHP_ of 2.0% PAAN, PAAND and PAANS in Na-MMT-based drilling fluid was investigated after thermal aging tests, and the results are shown in Fig. 6 and 7. With the increase of temperature, the FL_API_ and FL_HTHP_ of Na-MMT/PAAN, Na-MMT/PAAND and Na-MMT/PAANS systems increased significantly. At the same temperature, the order of controlling FL_API_ and FL_HTHP_ was PAAND > PAANS > PAAN in Na-MMT/PAANS-based drilling fluid. When the temperature was higher than 200 °C and 220 °C, the filtration of the Na-MMT/PAAN and Na-MMT/PAANS systems was out of control, respectively. In contrast, the FL_API_ and FL_HTHP_ of the Na-MMT/PAAND system were 10.0 mL and 22.0 mL after thermal aging tests at 230 °C, and when the thermal aging temperature rose to 240 °C, the FL_API_ and FL_HTHP_ of Na-MMT-based drilling fluid treated with PAAND increased to 12.0 mL and 30.0 mL, respectively, which was still within the range of control. There is sufficient experimental evidence to prove that the temperature resistance of PAAND as a fluid loss additive can reach 240 °C. Therefore, based on the above results, it is clear that the introduction of rigid monomers, especially in the backbone, can effectively improve the temperature resistance of polymeric fluid loss additives.

**Fig. 8:**
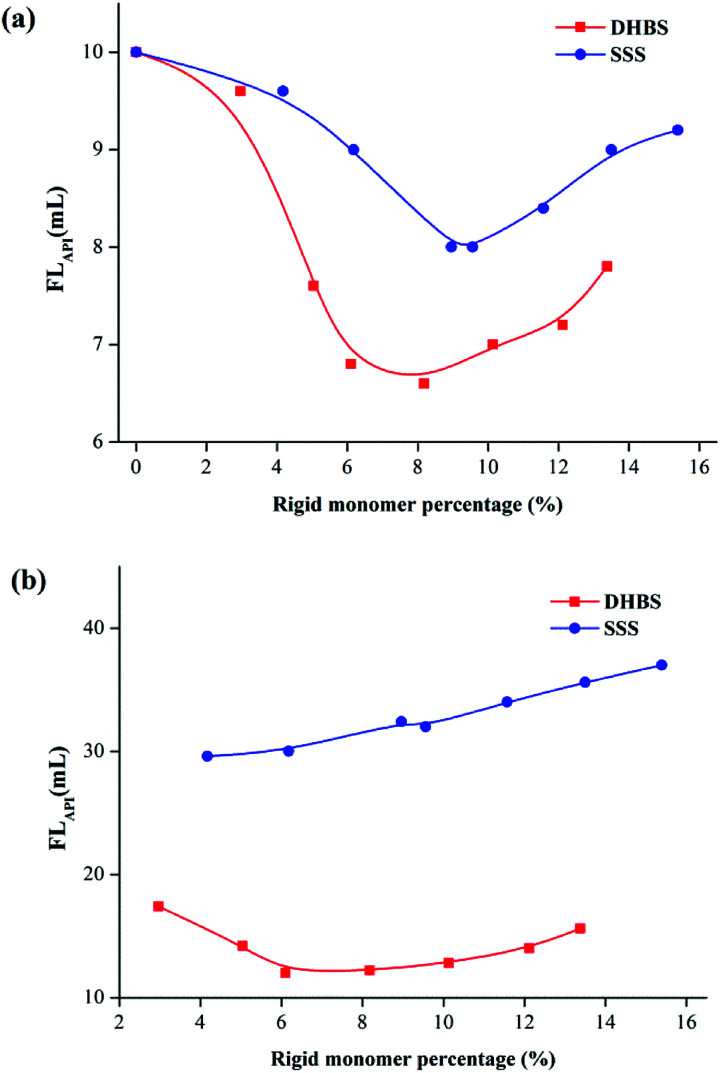
Effect of rigid monomer percentage in polymer molecules on FL_API_ in Na-MMT drilling fluid after thermal aging tests at (a) 200 °C and (b) 240 °C.

**Fig. 9:**
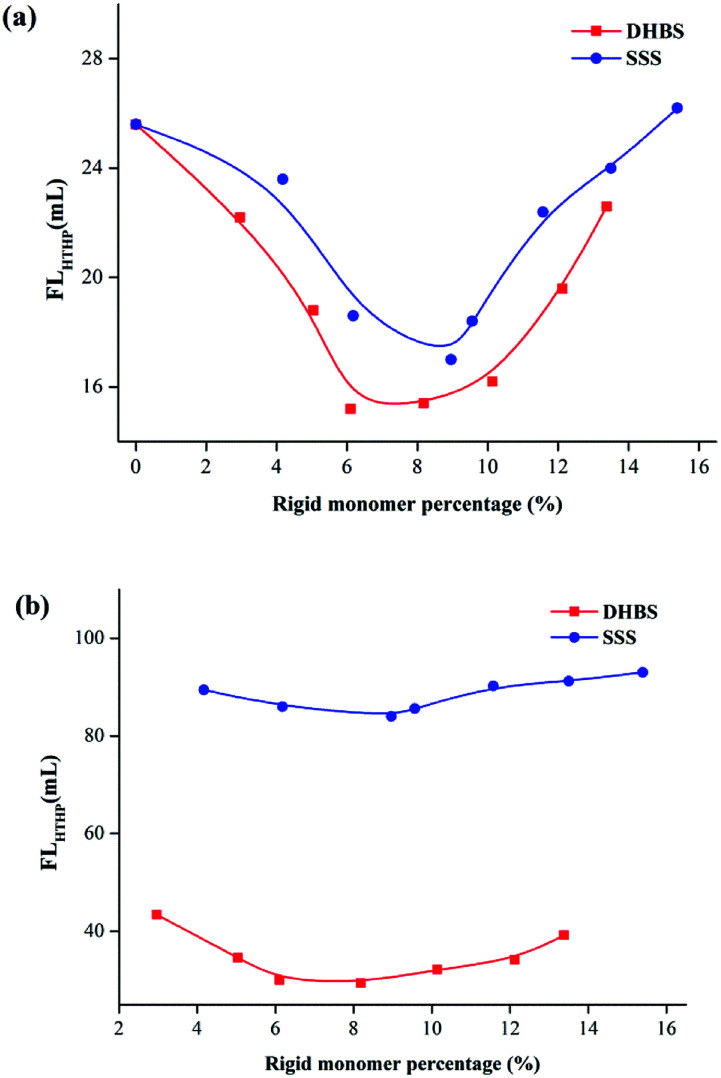
Effect of rigid monomer percentage in polymer molecules on FL_HTHP_ in Na-MMT drilling fluid after thermal aging tests at (a) 200 °C and (b) 240 °C.

Fig. 8a and b illustrate the FL_API_ curves of 2.0% PAAND-type and PAANS-type series of polymers in Na-MMT-based drilling fluid after thermal aging tests at 200 °C and 240 °C, respectively. When the thermal aging temperature was 200 °C, the FL_API_ of PAADN and PAANS in Na-MMT-based drilling fluid was significantly affected by the percentage of rigid monomers. When the percentage of rigid monomers in polymer molecules was 6.0–8.0%, the FL_API_ was in a low range. When the thermal aging temperature reached 240 °C, the FL_API_ of the PAAND-type series of polymers was significantly affected by the percentage of rigid monomers compared with that of the PAANS-type series of polymers. As indicated in Fig. 9a and b, which illustrate the FL_HTHP_ curves of 2.0% PAAND-type and PAANS-type polymers in Na-MMT-based drilling fluid after thermal aging tests at 200 °C and 240 °C, the variation trend of FL_HTHP_ with the percentage of rigid monomers is similar to that of FL_API_ with the percentage of rigid monomers. The polymeric fluid loss additive with rigid monomers in the polymer molecules can improve the filtration properties of water-based drilling fluid, especially when rigid monomers are introduced into the backbone. However, the introduction ratio must be appropriate. An excessive ratio will reduce the percentage of other functional groups and affect the performance of the fluid loss additive.

### Performance sustainability

**Fig. 10:**
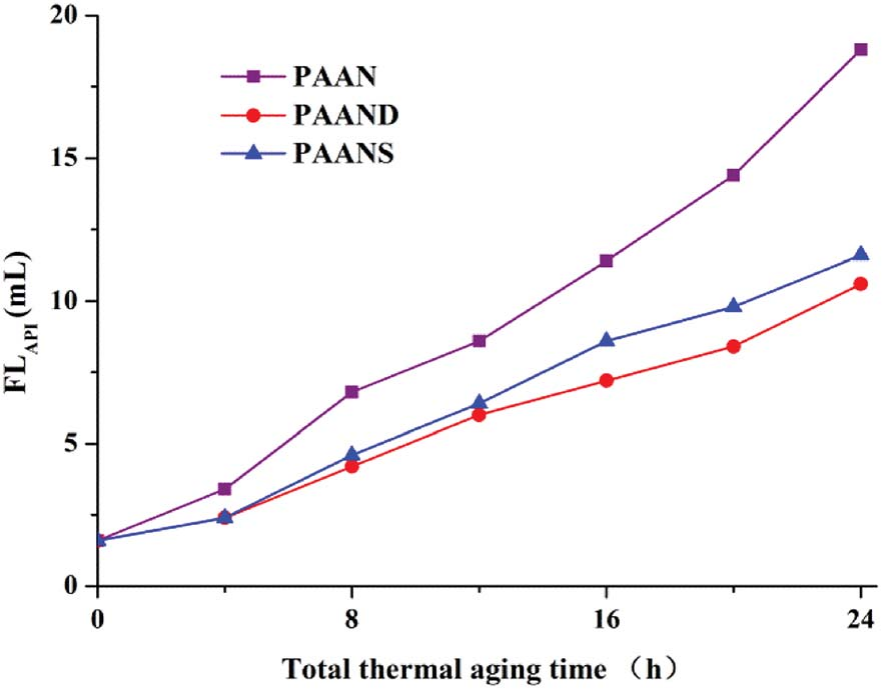
FL_API_ of PAAN, PAAND and PAANS in Na-MMT-based drilling fluid after multiple shearing and thermal aging tests.

**Fig. 11:**
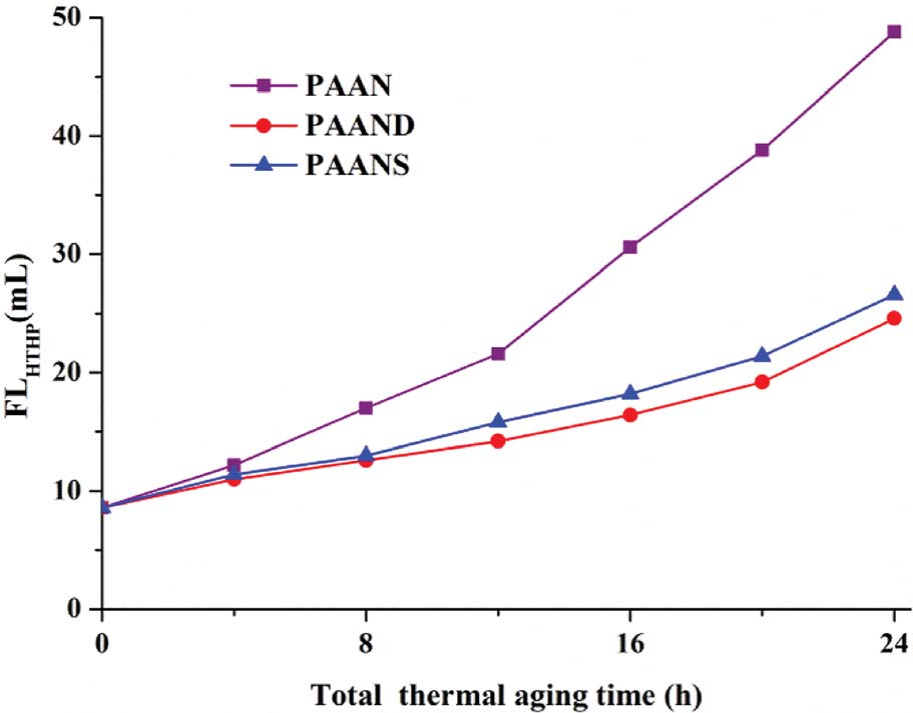
FL_HTHP_ of PAAN, PAAND and PAANS in Na-MMT-based drilling fluid after multiple shearing and thermal aging tests.

Fig. 10 and 11 illustrate the effects of the total thermal aging time on the FL_API_ and FL_HTHP_ of PAAN, PAAND and PAANS in Na-MMT-based drilling fluid. As indicated in Fig. 10 and 11, with the increase of the total thermal aging time, the FL_API_ and FL_HTHP_ of Na-MMT/PAAN, Na-MMT/PAAND and Na-MMT/PAANS increased significantly, indicating that shear forces and high temperature have certain negative effects on the filtration performance of the polymeric fluid loss additives. The FL_API_ of PAAN, PAAND and PAANS increased from 1.6 mL before thermal aging to 18.8 mL, 10.6 mL and 11.6 mL after 24 h of thermal aging, respectively. The FL_HTHP_ of PAAN, PAAND and PAANS increased from 8.6 mL before the thermal aging tests to 48.8 mL, 24.6 mL and 26.6 mL, respectively, when the total thermal aging time was 24 h. Moreover, at the same total thermal aging time, the control order of FL_API_ and FL_HTHP_ was PAAND > PAANS > PAAN in drilling fluid, indicating that the introduction of rigid monomers in the molecular chain, especially the polymer backbone, is helpful for improving the performance sustainability of polymeric fluid loss additives. This may be because the introduction of rigid monomers in the molecular chain can hinder the rotation of chemical bonds and conformational changes, thus reducing the thermal motion speed of the molecular chain and increasing its stability at high temperature. In particular, the introduction of rigid monomers into the backbone can effectively improve its activity at high temperature, increase the shear and high temperature resistance of the molecular chain, and achieve the goal of performance sustainability.

### Mechanism analysis

**Fig. 12:**
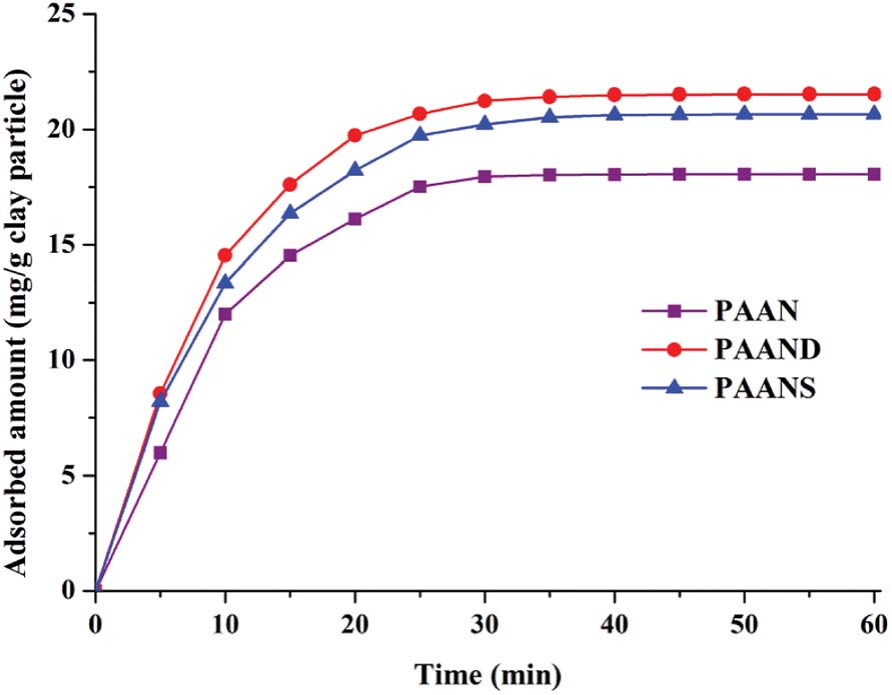
Dynamic adsorption curves of PAAN, PAAND and PAANS in Na-MMT-based drilling fluid.

The adsorption capacities of PAAN, PAAND and PAANS at different temperatures were measured to investigate the effect of rigid monomers on the improvement of interaction intensity between Na-MMT and the polymers. The dynamic adsorption curves of PAAN, PAAND and PAANS at 160 °C are shown in Fig. 12.

As indicated in Fig. 12, the adsorption reaction of PAAN, PAAND and PAANS in Na-MMT-based drilling fluid reached equilibrium after 30 min, and the equilibrium adsorption concentration was 18.06 mg g^−1^ clay particles, 21.53 mg g^−1^ clay particles and 20.66 mg g^−1^ clay particles, respectively. It was clearly found that the adsorbed amounts of PAAND and PAANS were similar but were obviously higher than that of PAAN at any moment, meaning that the rigid monomer can promote polymer adsorption on Na-MMT to a certain extent.

**Fig. 13:**
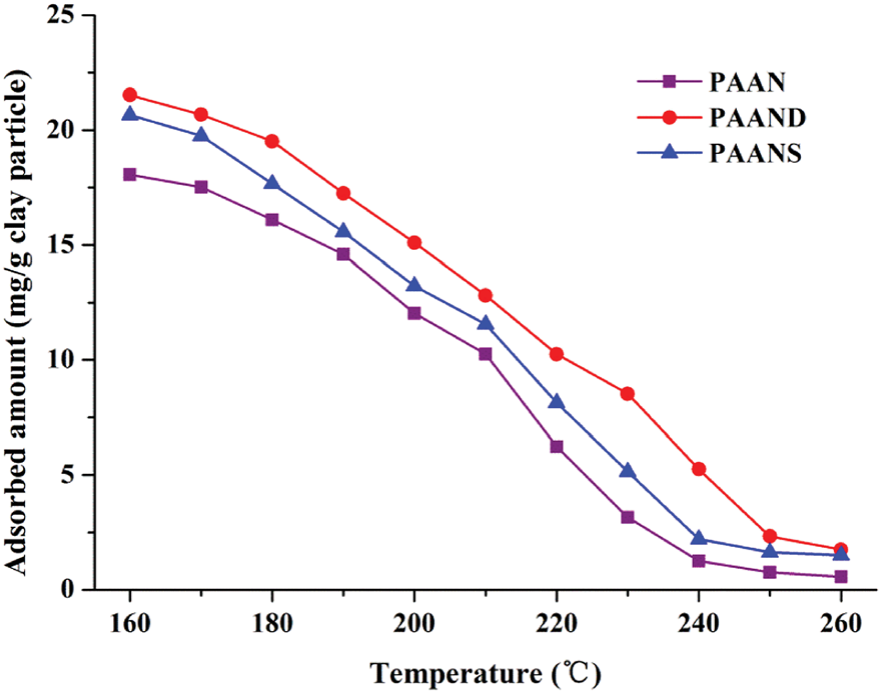
Effect of temperature on the adsorption capacity of PAAN, PAAND and PAANS.

To further investigate the effect of rigid monomers in polymers used as fluid loss additives, especially at high temperature, a comparative test between PAAN, PAAND and PAANS was carried out in Na-MMT/PAAN, Na-MMT/PAAND and Na-MMT/PAANS systems at a concentration of 1.0 wt% after thermal aging tests for 16 h. The adsorption curves of PAANS and PAAN at temperatures from 160 °C to 260 °C are shown in Fig. 13.

As indicated in Fig. 13, the adsorbed amount of PAAN, PAAND and PAANS in Na-MMT-based drilling fluid decreased with increasing temperature. The adsorbed amount of PAAND was 21.53 mg g^−1^ clay particles at 160 °C. With a temperature increase to 260 °C, the adsorbed amount of PAAND dropped to 1.75 mg g^−1^ clay particles. Comparatively, the adsorbed amount of PAAN reduced from 18.06 mg g^−1^ clay particles to 0.56 mg g^−1^ clay particles, and the adsorbed amount of PAANS reduced from 20.66 mg g^−1^ clay particles to 1.50 mg g^−1^ clay particles when the temperature was increased from 160 °C to 260 °C, respectively. Obviously, the order of adsorbed amount was PAAND > PAANS > PAAN in the whole temperature range investigated, indicating a stronger interaction between Na-MMT and polymeric fluid loss additives containing rigid monomers. A possible reason for this is that rigid monomers in a polymer molecule can reduce the thermal movement of molecular chains at high temperature, which helps to reduce desorption between the molecular chains and Na-MMT at high temperature.

**Fig. 14:**
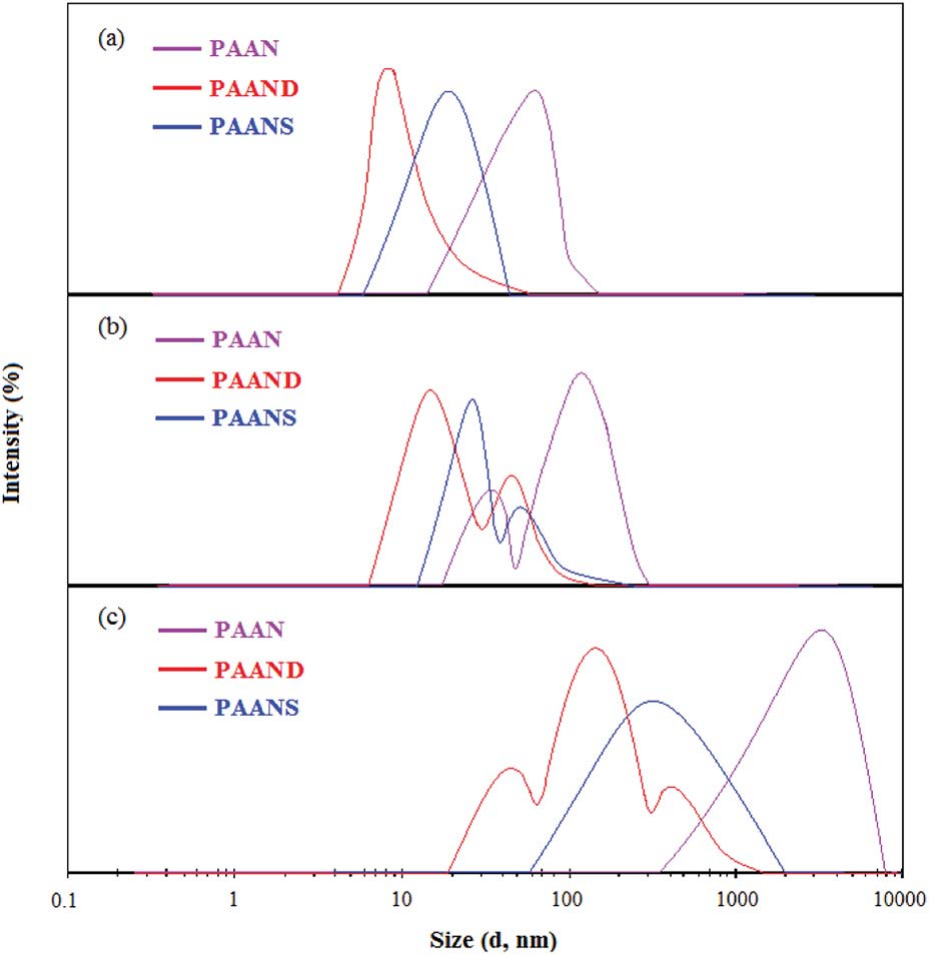
The curves of particle size distribution of PAAN, PAAND and PAANS in Na-MMT-based drilling fluid after thermal aging tests at (a) 160 °C, (b) 200 °C and (c) 240 °C.

The particle size distributions of PAAN, PAAND and PAANS in Na-MMT-based drilling fluid are shown in Table 6 and Fig. 14. As indicated in Table 6 and Fig. 14, with increasing temperature, the *D*_10_, *D*_50_, *D*_90_ and *D*_av_ of PAAN, PAAND and PAANS in Na-MMT-based drilling fluid increased, and the specific surface area (SSA) decreased significantly.

**Table 6:** Effect of thermal aging temperature on the particle size distribution of PAAN, PAAND and PAANS in Na-MMT-based drilling fluid

Fluid formulation	Thermal aging temperature (°C)	*D*_10_ (nm)	*D*_50_ (nm)	*D*_90_ (nm)	*D*_av_ (nm)	SSA (m^3^ kg^−1^)
Na-MMT-based drilling fluid + 2.0% PAAN	160	16.6	78.0	100.5	83.2	65 355.9
	200	21.3	104.7	116.7	109.6	55 771.8
	240	512.8	2704.2	6333.1	2806.0	2016.7
Na-MMT-based drilling fluid + 2.0% PAAND	160	8.7	14.8	23.4	17.3	195 050.8
	200	12.6	22.4	86.5	23.0	126 571.3
	240	33.7	106.6	668.2	110.9	7312.5
Na-MMT-based drilling fluid + 2.0% PAANS	160	12.6	20.5	27.2	21.0	14 416.0
	200	24.1	31.7	100.0	32.0	9179.4
	240	91.2	365.9	1045.3	379.6	3498.1

The curve of particle size distribution of PAAN and PAANS changed from having single peak at 160 °C to having two peaks at 200 °C, which then turned into a single peak again at 240 °C. The curve of the particle size distribution for PAAND changed from having a single peak at 160 °C to two peaks at 200 °C, and then three peaks 240 °C. The curves indicate a gradual transition towards larger particle sizes, meaning that high temperatures can improve the coalescence of Na-MMT particles. The main reason for this is that high temperature promotes desorption between the polymer and Na-MMT particles, and the Na-MMT particles collide with each other at high temperature and then coalesce. It is also worth noting that polymeric fluid loss additives with rigid monomers, especially in the polymer backbone, are better able to block the transition of Na-MMT particles to large particle sizes in Na-MMT-based drilling fluid. The reason for this phenomenon is that the polymeric fluid loss additive with rigid monomers exhibits strong adsorption on the Na-MMT particle surface and can form a thick hydration film, which effectively restrains the coalescence effect of Na-MMT particles at high temperature.

**Fig. 15:**
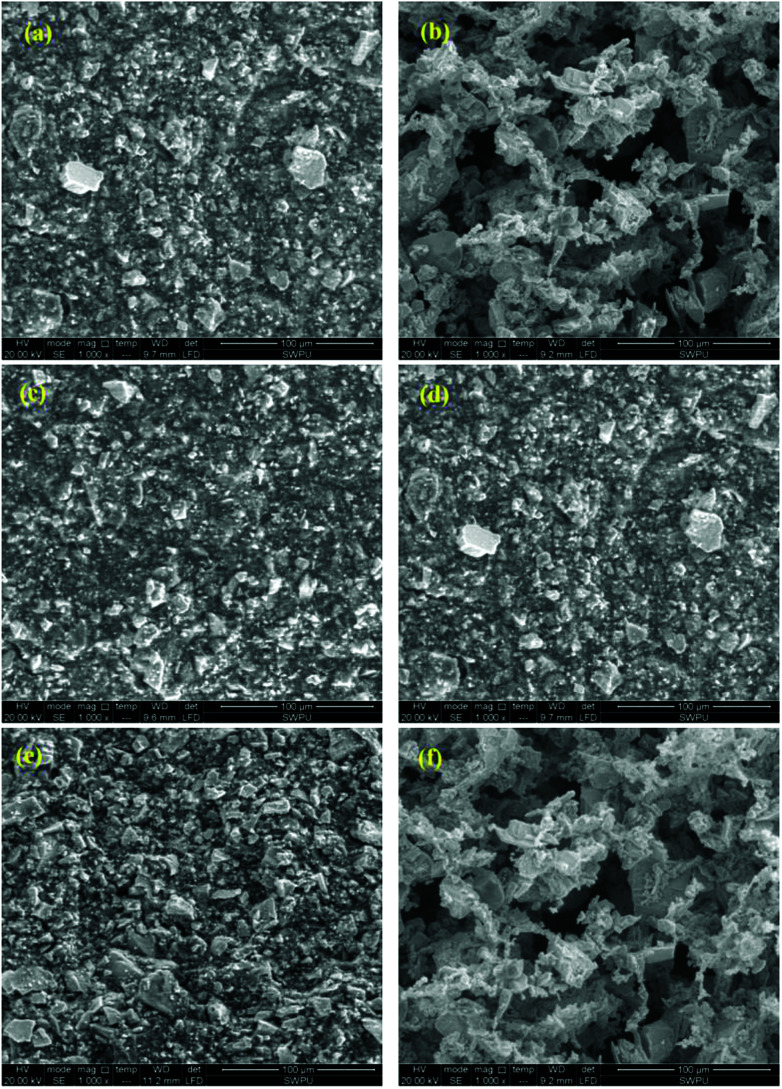
Filter cake morphologies of PAAN, PAAND and PAANS in Na-MMT-based drilling fluid after thermal aging tests at different temperatures: (a) PAAN, 200 °C; (b) PAAN, 240 °C; (c) PAAND, 200 °C; (d) PAAND, 240 °C; (e) PAANS, 200 °C; (f) PAANS, 240 °C.

Filter cakes of PAAN, PAAND and PAANS in Na-MMT-based drilling fluid show various morphologies in Fig. 15. Evident differences in the characteristics of the formed microstructures of the filter cakes can be observed. As indicated in Fig. 15a, c and e, the filter cakes of PAAN, PAAND and PAANS in Na-MMT-based drilling fluid were smooth and uniform on their surfaces after thermal aging at 200 °C. The Na-MMT particles were wrapped by polymer, and the accumulation was relatively dense. There were no significant gullies or pores on the surface of these filter cakes. In contrast, when the thermal aging temperature changed to 240 °C, the surface microstructures of the filter cakes of PAAN, PAAND and PAANS in Na-MMT-based drilling fluid were very different. In comparison to Fig. 15a, Fig. 15b shows that the filter cake surface of PAAN was uneven and friable, with some ravines and pores. Several big Na-MMT particles could be seen, so the Na-MMT-based drilling fluid treated with PAAN exhibited poor dispersion and the filter cake formed was of a poor quality, causing terrible dehydration. In Fig. 15d, although a certain number of large Na-MMT particles appear on the filter cake surface of Na-MMT-based drilling fluid treated with PAAND, small Na-MMT particles are still distributed, and there are no obvious gullies and pores, indicating that the filter cake had a lower permeability and was of better quality after thermal aging at 240 °C. Compared with Fig. 15e, the polymer PAANS on the surface of the filter cake shown in Fig. 15f is obviously curled, and there are more large Na-MMT particles stacked up. The surface is uneven and friable, and there are larger pores in the surface. These results indicate that the filter cake of the Na-MMT-based drilling fluid treated with PAANS had a high permeability and had lost the ability to control its filtration properties after thermal aging at 240 °C.

An evaluation of the filter cake morphology results shows that the use of polymers with rigid monomers in the backbone as fluid loss additives can effectively keep the fine particle size dispersion in the drilling fluid and improve the filter cake quality. It is of great significance to improve the filtration properties of water-based drilling fluid.

## Conclusions

In this study, the effect of molecular flexibility on the rheological and filtration properties of synthetic polymers as fluid loss additives has been investigated in water-based drilling fluid. Three kinds of polymeric fluid loss additives (PAAN, PAAND and PAANS) were synthesized using HRP as a catalyst under the same conditions. PAAN does not contain any rigid monomers. PAAND is a kind of synthetic polymer with phenyl groups in the backbone, while PAANS is a kind of synthetic polymer with phenyl groups in the side chains. The Na-MMT-based drilling fluids treated with PAAN, PAAND and PAANS presented rheological properties, filtration properties and performance sustainability within the specifications recommended by the American Petroleum Institute. Compared with flexible polymeric fluid loss additives, PAAN, PAAND and PAANS with rigid monomers exhibit better rheological properties, filtration properties and performance sustainability in water-based drilling fluid. In particular, the high temperature resistance of PAAND can reach 240 °C, which indicates that the temperature resistance of a polymeric fluid loss additive can be improved by introducing rigid monomers into the backbone of the polymer molecule to improve its rigidity.

Mechanistic investigations revealed that the improvement is due to the higher adsorption of synthetic polymers containing rigid monomers as fluid loss additives, especially those with rigid monomers introduced into the polymer backbone. This high affinity ensures that polymeric fluid loss additives can strongly encapsulate Na-MMT particles at high temperature, form hydration films on the Na-MMT particle surface and reduce the tendency of Na-MMT particles to collide with each other to form large particles. It can help to maintain a certain number of small Na-MMT particles in the Na-MMT-based drilling fluid, fill the pores in the surfaces of filter cakes and reduce the permeability and improve the quality of filter cakes, to control the loss of water from drilling fluid.

## Conflicts of interest

There are no conflicts to declare.

## Supplementary Material

## References

[cit1] Peer D. V. , D'Hawse F. C. and Dams R. J., Additive to reduce fluid loss for drilling fluid, *US Pat.*, 2013/8343895 B2, 2013

[cit2] Sadeghalvaad M., Sabbaghi S. (2015). Powder Technol..

[cit3] Fink J. K. , Petroleum Engineer's Guide to Oil Field Chemicals and Fluids, Gulf Professional Publishing, Texas, 2012

[cit4] Khodja M., Canselier J. P., Bergaya F., Fourar K., Cohaut N., Benmounah A. (2010). Appl. Clay Sci..

[cit5] Wu Y. M., Zhang B. Q., Wu T., Zhang C. G. (2001). Colloid Polym. Sci..

[cit6] Quan H. P., Li H., Huang Z. Y., Zhang T. L., Dai S. S. (2014). Int. J. Polym. Sci..

[cit7] Falose O. A., Ehinola O. A., Nebeife P. C. (2008). Appl. Clay Sci..

[cit8] Kelessidis V. C., Tsamantaki C., Michalakis A., Christidid G. E., Makri P., Papanicolaou K., Foscolos A. (2007). Fuel.

[cit9] Li M. C., Wu Q. L., Song K. L., Qing Y., Wu Y. Q. (2015). ACS Appl. Mater. Interfaces.

[cit10] Lin L., Luo P. (2015). J. Appl. Polym. Sci..

[cit11] Lin L., Luo P. (2018). Appl. Clay Sci..

[cit12] Zhang L. M., Tan Y. B., Li Z. M. (1999). Colloid Polym. Sci..

[cit13] Dias F. T. G., Souza R. R., Lucas E. F. (2015). Fuel.

[cit14] Salami O. T., Plank J. (2012). J. Appl. Polym. Sci..

[cit15] Ahmad H. M., Kamal M. S., AI-Harthi M. A. (2018). J. Mol. Liq..

[cit16] Bai X. D., Yang Y., Xiao D. Y., Pu X. L., Wang X. (2015). J. Appl. Polym. Sci..

[cit17] Cao J., Meng L. W., Yang Y. P., Zhu Y. J., Wang X. Q., Yao C. Y., Sun M. B., Zhong H. Y. (2017). Energy Fuels.

[cit18] Plank K., Brandl A., Lummer N. R. (2007). J. Appl. Polym. Sci..

[cit19] Hill R., Walker E. E. (1948). J. Polym. Sci..

[cit20] Kim S. I., Ree M., Shin T. J., Jung J. C. (1999). J. Polym. Sci., Part A: Polym. Chem..

[cit21] Mohamed N. A. (1994). Polym. Degrad. Stab..

[cit22] Liaw D. J., Chang F. C., Leung M. K., Chou M. Y., Muellen K. (2005). Macromolecules.

[cit23] Chen T. A., Jen A. K. Y., Cai Y. M. (1995). J. Am. Chem. Soc..

[cit24] Varganici C. D., Rosu D., Mic C. B., Rosu L., Popovici D., Hulubei C., Simionescu B. C. (2015). J. Anal. Appl. Pyrolysis.

[cit25] Gu Y., Sun Z., Gong S. M., Zhang H., Gong Q., Liu L. L., Wang Y. H. (2015). J. Mater. Sci..

[cit26] Mir A. A., Wagner S., Kramer R. H., Deglmann P., Emrick T. (2016). Polymer.

[cit27] Liu W., Luo X., Bao Y., Liu Y. P., Ning G. H., Abdelwahab I., Li L. J., Nai C. T., Hu Z. G., Zhao D., Liu B., Quek S. Y., Loh K. P. (2017). Nat. Chem..

[cit28] Wan T., Yao J., Sun Z. S., Wang L., Wang J. (2011). J. Pet. Sci. Eng..

[cit29] Ma X. P., Zhu Z. X., Shi W., Hu Y. Y. (2016). Colloid Polym. Sci..

[cit30] Chilingarian G. V., Alp E., Caenn R., Salem M. A., Uslu S., Gonzales S., Dorovi R. J., Mathur R. M., Yen T. F. (1986). Energy Sources.

[cit31] Dairanieh I. S., Lahalih S. M. (1988). Eur. Polym. J..

[cit32] Chu Q., Luo P. Y., Zhao Q. F., Feng J. X., Kuang X. B., Wang D. L. (2013). J. Appl. Polym. Sci..

